# Modulating stress and sleep through a multi-compound dietary supplement: a randomized, double-blind, placebo-controlled trial

**DOI:** 10.1080/15502783.2026.2740227

**Published:** 2026-10-04

**Authors:** Gianna F. Mastrofini, Riccardo F. Romersi, Maria G. Lombardi, Katelynn T. Persaud, Monica M. Ryan, Katie R. Hirsch, Amy L. Fraley, J. Jay Dawes, Sten O. Stray-Gundersen, James W. Hardin, Shawn M. Arent

**Affiliations:** a Arnold School of Public Health, University of South Carolina, Department of Exercise Science, Columbia, SC, United States; b School of Kinesiology, Oklahoma State Univeristy, Department of Applied Health and Recreation, Stillwater, OK, United States; c Arnold School of Public Health, University of South Carolina, Department of Epidemiology and Biostatistics, Columbia, SC, United States

**Keywords:** Adaptogens, mood, sleep disturbance, perceived stress, sleep quality

## Abstract

**Background:**

Stress and poor sleep are prevalent public health concerns that negatively impact well-being and daily functioning. Although adaptogens and sleep-focused supplements have separately demonstrated potential benefits, few studies have examined combined effects, particularly in healthy populations. Therefore, this study evaluated the impact of morning adaptogens, evening sleep-focused supplements, and their combination on stress, mood, and subjective and objective sleep quality.

**Methods:**

Using a double-blind, placebo-controlled design, 103 healthy physically active adults (F = 53; age = 34.8 ± 9.6 yrs; BMI = 24.0 ± 2.7 kg/m^2^) were randomized to one of four treatment groups: 1) AMPM (*n* = 25): active morning (AM) and active evening (PM); 2) AM (*n* = 26): active AM and placebo PM; 3) PM (*n* = 25): placebo AM and active PM; and 4) PL (*n* = 27): placebo AM and placebo PM). The AM group contained 600 mg ashwagandha and 300 mg *Rhodiola rosea (R. rosea)*. The PM group contained 2 g Magnesium L-threonate (MgT) (delivering 145 mg of magnesium), 200 mg L-theanine, and 50 mg apigenin. The total study duration was four weeks, consisting of one baseline week, two weeks of supplementation, and one washout week. Participants completed daily stress questionnaires and weekly questionnaires on perceived stress, affect, and subjective sleep (Patient-Reported Outcome Measurement Information System (PROMIS), and monthly subjective sleep (Pittsburgh Sleep Quality Index (PSQI) at baseline and post-washout. Objective sleep data were collected nightly using a wearable sleep monitoring device. Data were analyzed using baseline-adjusted linear mixed-effects models with alpha set at 0.05. Tukey-adjusted post hoc comparisons were performed, and effect sizes are reported as partial eta squared (η^2^) and Cohen's d (*d*).

**Results:**

The AM group showed a decrease in weekly perceived stress (*p* = 0.034; η^2^ = 0.020), whereas there were no differences in daily stress (*p *< 0.05). For positive affect, there was a significant effect in the AM group at the end of the cessation period (*p* = 0.003; *d* = 1.68), which continued to increase, whereas the AMPM group showed a decrease (*p* = 0.009; *d* = 0.16). The AM group showed a decrease in subjective sleep-related impairments (*p* = 0.032, *d* = 2.74) and in objective wake after sleep onset (*p* = 0.047, η^2^ = 0.020). PSQI subjective sleep quality was significantly improved in the AM group compared with the AMPM group (mean difference = 0.66 AU, *p *< 0.001, *d* = 1.45).

**Conclusions:**

Morning supplementation with adaptogens (ashwagandha and *R. rosea)* was associated with improvements in subjective stress, sleep quality, and positive affect following 14 days in healthy physically active adults as compared to the placebo product. Minimal effects on other outcomes may reflect limited benefits in healthy individuals over a short supplementation period. Future research should investigate longer interventions and populations with greater baseline stress and sleep disturbance.

## 1. Introduction

It has been suggested that approximately half the global population experiences elevated levels of stress, attributed to workplace and economic stressors, manifested as depression and/or anxiety [1]. Furthermore, 30%–62% of adults report inadequate sleep [2,3]. Both chronic stress and poor sleep are associated with decrements in health, well-being, and daily occupational performance [4,5]. As a result, chronic stress and sleep deficiencies pose a major public health concern.

Stress and sleep deprivation are both related and interrelated. Acute sleep restriction increases hypothalamic-pituitary-adrenal (HPA) axis activity [6], and chronic sleep loss may alter HPA responsiveness [6,7]. Such dysregulation may perpetuate a cycle of hyperarousal and heightened stress reactivity, thereby exacerbating both poor sleep quality and stress. Poor sleep and chronic stress interact to create a self-reinforcing cycle of stress and insufficient recovery. Due to this bidirectional relationship, interventions that target both daytime stress modulation and sleep quality are likely to be more effective than interventions targeting either in isolation. Given the widespread prevalence and multifactorial consequences of these issues, there is increasing interest in safe, non-pharmacological strategies that can simultaneously mitigate stress and improve sleep quality.

One such approach involves adaptogens, a class of naturally occurring, plant-derived compounds proposed to enhance physiological resilience to stress [8]. Two widely studied adaptogens are *Withania somnifera* (ashwagandha) and *Rhodiola rosea (R. rosea),* both of which have been used for centuries in traditional Eastern medicine [9]. It has been proposed that ashwagandha may influence both stress and sleep through gamma-aminobutyric acid (GABA) related mechanisms and HPA axis modulation [10]. There is meta-analytic support for the effects of ashwagandha on reductions in perceived stress scale (PSS), negative mood, and serum cortisol following 4–13 weeks of supplementation with 250–500 mg/day in stressed populations [11–13]. Improvements have also been observed in objective stress markers, such as C-reactive protein, following 60 days of 125 mg ashwagandha supplementation [14]. Further improvements have been demonstrated in sleep outcomes, as measured by the Pittsburgh Sleep Quality Index Questionnaire (PSQI) and objective actigraphy in stressed and insomniac participants [15,16] and healthy adults [17]. Similar to ashwagandha, *R. rosea* is promoted for mitigating fatigue and increasing stress resilience [18]. Proposed mechanisms of action include inhibition of stress-activated protein kinase pathways and cortisol modulation during acute stress exposure [8]. A substantial body of research supports its effects in stressed populations, with doses of 170–576 mg daily of *R. rosea* showing improvements in concentration, mood, subjective stress, and morning cortisol [19,20]. While adaptogens such as ashwagandha and *R. rosea* primarily influence stress and sleep via the HPA axis and autonomic nervous system, combining them with complementary supplements may provide broader neurobiological support.

Micronutrients (e.g. magnesium L-threonate), amino acids (e.g. L-theanine), and flavonoids (e.g. apigenin) all act on physiological systems related to sleep quality and stress modulation. Magnesium plays a central role in hundreds of enzymatic reactions, including the regulation of glutamatergic and GABAergic signaling, the synthesis of a precursor to melatonin, and cortisol reduction [21,22]. Although dietary magnesium is associated with better sleep quality [23], research on magnesium supplementation has yielded mixed results, likely due to heterogeneity in doses, populations studied, and formulations [24,25]. Importantly, magnesium L-threonate (MgT) more readily crosses the blood-brain barrier and may therefore more effectively target neurobiological-related deficiencies compared with other forms of magnesium [26]. A 21-day trial with 75 mg/day of MgT in participants with sleep issues showed improvements in objective measures of deep sleep and mood [27]. L-theanine, an amino acid naturally found in green tea, also demonstrates positive effects on stress and sleep in both animal and human studies [28,29] by modulating multiple neurotransmitter systems including dopamine, serotonin, glutamate, glutamine, and GABA [30,31]. Acute supplementation of 200 mg L-theanine reduced the stress response during an experimental stress task [32], while four weeks of 200 mg supplementation reduced subjective stress and subscales of the PSQI [33]. Further, 400 mg supplementation for one week prior to a high-stress academic period decreased salivary alpha-amylase and perceived stress in university students [34]. Meta-analytic results support improvements in subjective sleep measures, although objective sleep metrics show mixed outcomes [28]. Apigenin, a naturally occurring plant flavonoid often studied as part of chamomile extract, may further support stress reduction and sleep improvement through GABAergic enhancement and modulation of cortisone, serotonin, and brain-derived neurotrophic factor levels [35,36]. Clinical studies, primarily in symptomatic populations, demonstrate improvements in mood, anxiety, and sleep quality across 200–1500 mg of chamomile extract or tea [37,38].

While each ingredient independently demonstrates potential benefits, combining them may provide synergistic improvements in stress responsivity and sleep regulation. Despite widespread consumer use of such multi-ingredient supplements, most studies evaluate single compounds in isolation or existing products with proprietary blends and underdosed ingredients [39,40]. Few trials assess both stress and sleep concurrently, and interventions aimed at modulating daytime stress and evening sleep quality may lead to significant improvement. There is a lack of studies incorporating both subjective and objective outcomes, especially in non-clinical populations, leaving a gap for an efficaciously dosed combination product in healthy adults. Therefore, this study aimed to evaluate the effects of a combined supplement containing ashwagandha, *R. rosea*, MgT, L-theanine, and apigenin on perceived stress and sleep quality in healthy physically active adults.

## 2. Methodology

### 2.1. Experimental approach

A randomized, double-blind, placebo-controlled design was utilized to assess the impact of a supplement blend on stress and sleep in healthy, physically active adults. Eligible participants were stratified by sex and block randomized into one of four study groups: 1) active morning (AM) and active evening (PM) (AMPM); 2) active AM and placebo PM (AM); 3) placebo AM and active PM (PM); and 4) placebo AM and placebo PM (PL). All study-related participation was conducted virtually over one month. Interested individuals were emailed a prescreening survey to establish preliminary eligibility. Participants who met the prescreening eligibility criteria participated in a call with a research team member to verify eligibility and review the study design and informed consent. Inclusion criteria were as follows: physically active (defined as ≥ 150 min/wk for ≥6 months, including potential combinations of resistance and/or aerobic training), aged 18 to 50 years (inclusive), a self-reported body mass index of 18.5 to 29.9 kg/m^2^, not diagnosed with any sleep- or stress-related disorders, and not currently taking any of the investigational products. Individuals were excluded if they were diagnosed with a sleep- or stress-related disorder, were currently taking any of the investigational products, lived outside of the United States, or were taking any medications or supplements to manage stress or sleep.

After initial screening, those still interested and eligible signed a virtual informed consent and were randomized to one of the four study product combinations. Participants were educated on study product use, daily study diary entries, questionnaire completion, and use of the SleepImage (Austin, TX, US) hardware devices and the accompanying Absolute Rest (Denver, CO, US) application for sleep tracking. Study diaries and questionnaires were tracked through People Science Software. Study products and the SleepImage device were then shipped to participants. Investigators and participants remained blinded to study product assignments. Upon receiving their study materials, participants completed four weeks of study-related measures, with one week of baseline, two weeks of product consumption, and one washout week (Figure 1). Participants were instructed not to change their nutrition and physical activity habits across the intervention.

**Figure 1.**
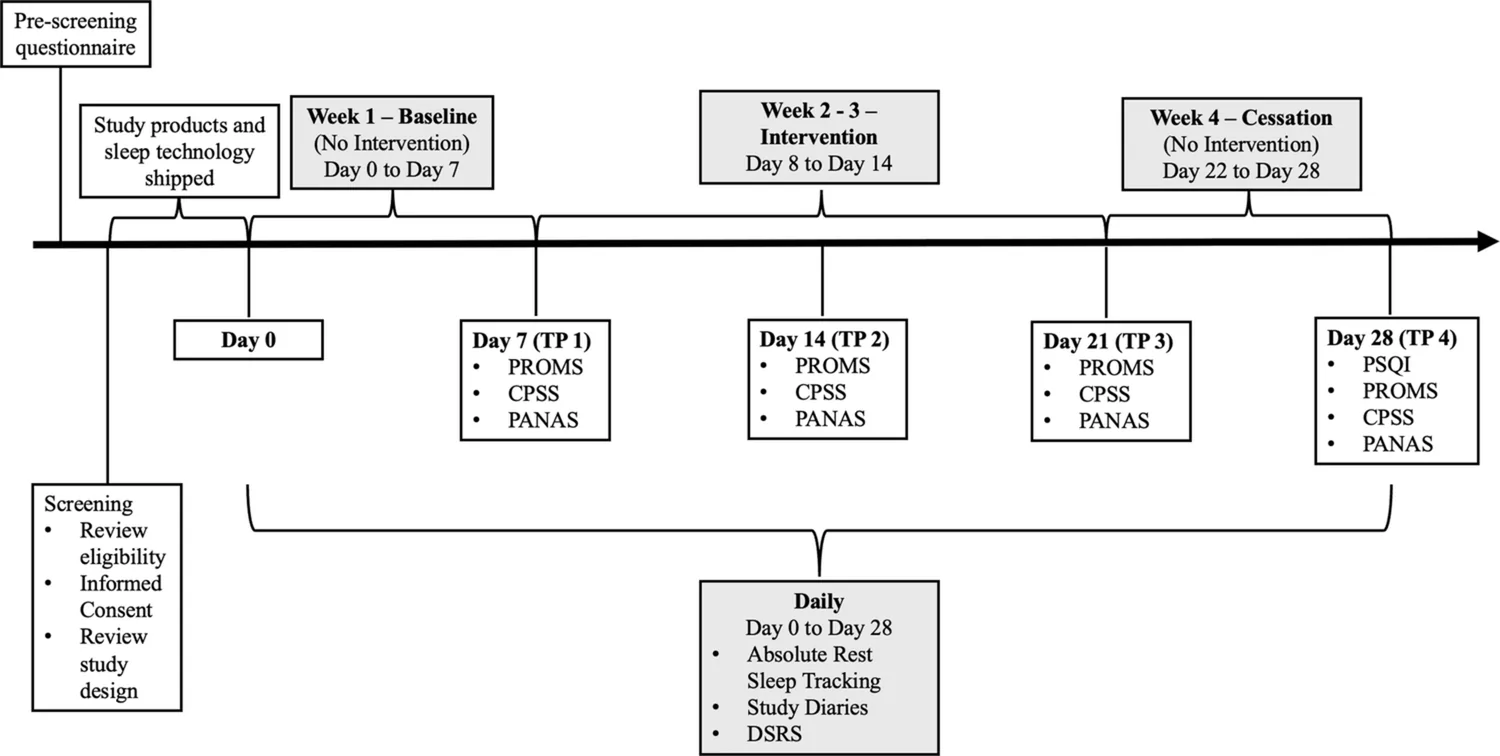
Study design flow chart.

This study was approved by the University of South Carolina Institutional Review Board, and informed consent was obtained for all participants (Pro00141237). It was also prospectively registered at ClinicalTrials.gov (NCT06889584).

### 2.2. Supplementation protocol

Study products were administered orally in capsule form, with 5 capsules taken in the morning and evening. The active AM product contained 600 mg ashwagandha (NooGandha^®^), standardized to 3.5% total withanolides and 300 mg *R. rosea*. (root extract), standardized to 3% rosavins and 2% salidroside. The active PM product contained 2 g Magnesium L-threonate (delivering 145 mg of magnesium) (Magtein^®^), 200 mg L-theanine, and 50 mg apigenin (from grapefruit). The placebo consisted of the same number of capsules as active supplements and was comprised of microcrystalline cellulose. Finished products were produced by Momentous LLC. All study supplement products were third-party tested by NSF Certified for Sport and/or Informed Sport as applicable. Participants were instructed to take their assigned products in the AM and PM at approximately the same time each day. Timing relative to meals was not controlled or standardized due to feasibility concerns. Daily compliance was monitored through online study diaries sent to participants every afternoon.

### 2.3. Participants

An *a priori* power analysis was conducted using G*Power (version 3.1) for which we specified an alpha level of 0.05 and a desired power of 0.80 for a repeated-measures ANOVA. This analysis indicated 24 participants per group would be needed (96 total). To account for a ~15% attrition rate, 115 individuals were randomized. A total of 543 individuals completed the initial screening questionnaire, and among them, 277 met the initial eligibility criteria and were contacted to be screened by a study team member (Figure 2). A total of 115 participants were randomized, and 103 participants finished the study. Participant descriptives and characteristics are contained in Tables 1 and 2.

**Table 1.** Baseline participant descriptive data.

	Sex	AMPM (*n* = 25; F = 12)	AM (*n* = 26; F = 14)	PM (*n* = 25; F = 12)	PL (*n* = 27; F = 15)
Age (years)	Overall	34.8 ± 9.6	35.0 ± 10.4	34.5 ± 9.8	34.9 ± 10
	Male	32.5 ± 7.2	34.4 ± 9.3	33.8 ± 10.5	38.3 ± 8.8
	Female	37.0 ± 9.1	35.6 ± 11.6	35.3 ± 9.7	32.2 ± 10.5
Reported BMI (kg/m^2^)	Overall	24.3 ± 2.5	24.1 ± 2.9	23.9 ± 2.5	23.8 ± 3.0
	Male	25.3 ± 2.6	26.4 ± 1.5	25.3 ± 2.3	24.3 ± 2.2
	Female	23.1 ± 1.9	22.2 ± 2.2	22.4 ± 1.8	23.3 ± 3.5
Physical activity (min)	Overall	392 ± 149	447 ± 193	386 ± 140	405 ± 157
	Male	455 ± 152	441 ± 205	439 ± 146	396 ± 169
	Female	323 ± 113	451 ± 189	328 ± 113	413 ± 152

Note: Data are presented as mean ± SD.

**Table 2.** Baseline participant characteristics.

Characteristic	AMPM	AM	PM	PL
**Race, *n* (%)**				
White	25 (100%)	23 (88%)	22 (88%)	22 (81%)
Asian	0 (0%)	1 (4%)	2 (8%)	3 (11%)
Black or African American	0 (0%)	1 (4%)	1 (4%)	1 (4%)
More than one race	0 (0%)	0 (0%)	0 (0%)	0 (0%)
Other/not reported	0 (0%)	1 (4%)	0 (0%)	1 (4%)
**Ethnicity, *n* (%)**				
Hispanic or Latino	1 (4%)	1 (4%)	1 (4%)	3 (11%)
Not Hispanic or Latino	24 (96%)	25 (96%)	24 (96%)	24 (88%)
Unknown/not reported	0 (0%)	0 (0%)	0 (0%)	0 (0%)

**Figure 2.**
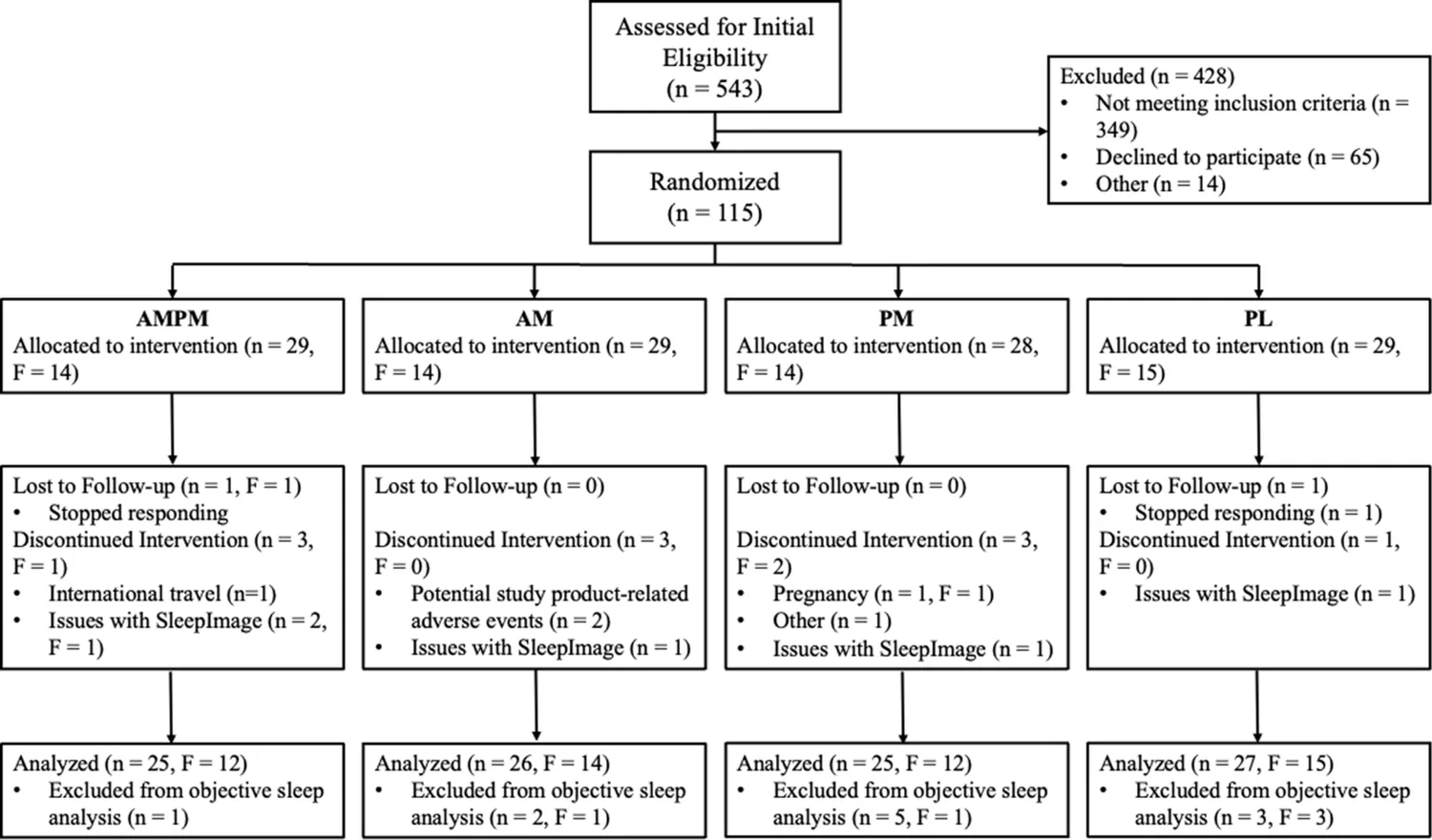
CONSORT diagram of study participants.

### 2.4. Study outcome measures

Cohen's perceived stress scale (PSS) [41], a 10-item questionnaire, was used to measure perceived stress. This scale has been used in prior research on adaptogens, supplements, and stress. Each question is rated on a five-point scale based on the frequency of each event. Anchors are 0 (“never”) and 4 (“often”). Half of the questions are positive and were reverse-scored. Results were then summed across questions with a maximum score of 40, indicating higher stress, and a minimum score of 0, indicating lower stress. Participants completed the PSS questionnaire on days 0, 7, 14, 21, and 28. The PSS-10 has been shown to have high reliability (Cronbach's ɑ ≥ 0.75 and ICC > 0.70) [42].

The daily stress response scale (DSRS) [43], a 30-item questionnaire, was used to assess both psychological and physiological reactions to daily stress using a 5-point Likert scale with anchors set at 0 (“never”) and 4 (“nearly all the time”). Higher scores indicate greater stress. Participants completed this questionnaire daily throughout the study. Data from weekly averages were used. The internal reliability of the DSRS is high (Cronbach's ɑ > 0.90) [43].

The positive and negative affect schedule (PANAS) [44], a 20-item questionnaire, was used to measure affect. Scores range from 10 to 50, with higher scores reflecting greater positive affect (PA) or negative affect (NA) for each respective subscale. Each question is scored on a 5-point Likert scale with anchors ranging from 1 (“very slightly or not at all”) and 5 (“extremely”). Participants completed the PANAS on days 0, 7, 14, 21, and 28. The PANAS has high reliability (Cronbach's ɑ > 0.75 and ICC > 0.80) [45,46].

The patient-reported outcomes measurement information system short form (PROMIS) [47] was used to assess both sleep disturbance and sleep-related impairments. Each questionnaire was comprised of 8 items, with higher scores indicating greater sleep disturbances or impairments. Raw questionnaire scores were converted to T-scores for analysis, with a population mean of 50 and a standard deviation of 10. Participants completed both PROMIS questionnaires on days 0, 7, 14, 21, and 28. Reliability (ICC) for both short forms ranges from 0.62 to 0.71 over multiple weeks [48] with consistent convergent validity [47].

Pittsburgh sleep quality index (PSQI) [49], a 19-item questionnaire, was used to assess sleep quality over the prior month; higher scores indicate greater sleep dysfunction with seven subscales encompassing subjective sleep quality, sleep latency, sleep duration, habitual sleep efficiency, sleep disturbances, use of sleep medication, and daytime dysfunction. Higher scores indicate worse sleep quality, with scores > 5 generally considered the threshold for classification of “poor sleep quality”. Participants completed the PSQI on days 0 and 28. It has an ICC of 0.65 [49].

Objective sleep tracking was measured using a SleepImage device in conjunction with the Absolute Rest analytics platform, enabling noninvasive, at-home nightly sleep monitoring. The SleepImage Ring continuously records pulse rate via photoplethysmography and wirelessly connects via Bluetooth to the Absolute Rest analytical application. Sleep metrics were derived using cardiopulmonary coupling (CPC) analysis, a validated physiological marker of sleep [50,51]. Participants wore the ring on their index finger and began the associated Absolute Rest application each night prior to sleep. The SleepImage device captured the original signals, and the Absolute Rest platform used proprietary algorithms applied to analyze the raw physiological data. Prior research has found high agreement between SleepImage and standard polysomnography [52]. The following items were assessed from the Absolute Rest reports: sleep quality index (SQI; a summary of sleep stability, fragmentation, and periodicity on a scale from 0—100), sleep efficiency (the ratio of total sleep divided by time in bed), sleep onset latency (the duration of time it takes to transition from wakefulness to sleep), sleep duration, total sleep time, wake after sleep onset (WASO; amount of time spent awake after initial sleep onset and prior to awakening) duration and times, and mean heart rate. Weekly averages were calculated for analysis, with a minimum of four nights of valid data required per week and at least two weeks of data required for participant inclusion.

#### 2.4.1. Daily study diaries

Participants completed all questionnaires and daily study diaries through a secure online research platform, Chloe (People Science Inc., West Hollywood, CA). Diaries were filled out once per day in the evening to denote supplement consumption and any adverse events or changes in health. At the end of weeks 2 and 3 of the supplement period, participants submitted pictures of their remaining study products. If compliance was below 80%, participants were re-educated on supplement consumption compliance. Re-education was delivered through email to the participant. After the first wave of 22 participants submitted their study product pictures, a mismatch emerged between the expected product remaining and the study diary entries. The research team realized there was a slight variation in the number of pills sent to each participant. To rectify this, after the first wave, participants were to send a baseline photo of their study products immediately upon receiving them. Thus, the compliance of 22 participants was evaluated using study diaries, and the compliance of the rest was evaluated using pill counts.

### 2.5. Statistical analysis

Data are presented as mean ± standard deviation (SD). One-way ANOVAs were performed on all variables to determine if there were baseline differences between groups. All participants who had >80% compliance throughout the study protocol were included in final analyses. Outcomes were analyzed as a 2 × 2 factorial design to evaluate the independent and combined effects of morning and evening supplementation. Two dichotomous treatment variables were created: AM condition, coded as active for participants receiving the AM product and placebo for those not receiving the AM product, and PM condition, coded as active for participants receiving the PM product and placebo for those not receiving the PM product.

For each outcome, baseline-adjusted linear mixed-effects models were used to examine post-baseline responses across the intervention. The model included AM condition, PM condition, timepoint, and all interaction terms as fixed effects, with the corresponding baseline value for the outcome included as a covariate. Age, body mass, and sex were included as additional covariates. Participant was included as a random intercept to account for repeated measures within individuals. Partial eta-squared (η^2^) values are reported for all omnibus tests. An alpha level of 0.05 was used to determine statistical significance. Cohen's d effect sizes were reported for all time and group main effects and any significant post-hoc interaction effects. Statistical analyses were performed using R (RStudio Version 4.5.2).

For objective sleep results, nightly outcome measures were averaged across each week, and to be included in the final analysis, participants needed to have ≥4 days of complete sleep data per week and ≥2 weeks of data. If participants had ≤4 days of data during the baseline week, they were re-educated on how to use the SleepImage device and repeated week 1. Data from the DSRS were also averaged weekly for the final analysis.

Due to incomplete data capture by the SleepImage device, 11 participants were excluded from the analysis of objective sleep metrics (Figure 2), thus results for objective data should be considered exploratory. Further, due to PSQI limitations of being administered following the cessation period, these results are exploratory.

## 3. Results

### 3.1. PSS

Age, weight, and sex were not significant predictors of perceived stress (*p* > 0.05). There was a significant AM effect (*p* = 0.034; η^2^ = 0.020). There was not a significant effect of PM (*p* = 0.684; η^2^ < 0.001) or AMPM (*p* = 0.279; η^2^ < 0.001). After the cessation week, the AM condition scores were worse than at TP 2 (*p* = 0.016; *d* = 0.61), while the AMPM group had improved PSS scores at this timepoint as compared to TP 2 (*p* = 0.012; *d* = 0.34) (Figure 3).

**Figure 3.**
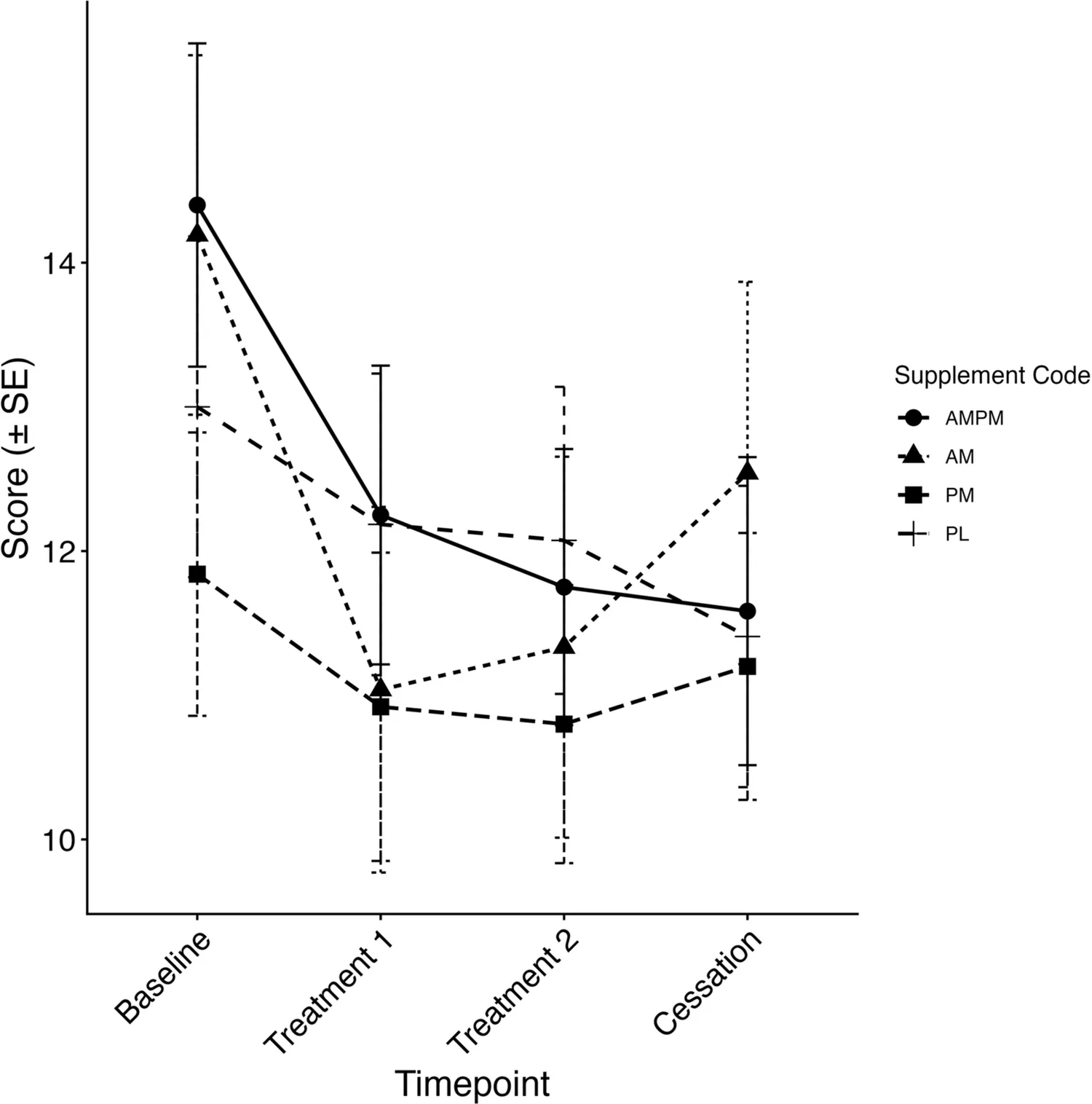
Group means ± standard error at each timepoint for the perceived stress scale questionnaire. The AM group improved over the intervention period compared with placebo (*p* = 0.034; η^2^ = 0.020). After the cessation week, the AM group scores were worse than at TP 2 (*p* = 0.016; d = 0.61), while the AMPM group had improved PSS scores at this timepoint as compared to TP 2 (*p* = 0.012; d = 0.34).

### 3.2. DSRS

Age, weight, and sex were not significant predictors of DSRS psychological or physiological scores (*p* > 0.05). None of the groups differed from placebo for DSRS psychological, AM (*p* = 0.805; η^2^ = 0.002), PM (*p* = 0.406; η^2^ = 0.002), and AMPM (*p* = 0.207; η^2^ = 0.021). None of the groups differed from placebo for DSRS physiological, AM (*p* = 0.232; η^2^ = 0.032), PM (*p* = 0.960; η^2^ < 0.001), and AMPM (*p* = 0.987; η^2^ = 0.002). Table 3 presents the unadjusted mean and standard deviation for both DSRS scales.

**Table 3.** Daily stress response survey by group and time.

Group	Baseline	Treatment 1	Treatment 2	Cessation
**Psychological**				
AMPM	8.0 ± 6.2	5.6 ± 5.1	4.6 ± 4.5	4.8 ± 5.4
AM	8.8 ± 8.0	6.7 ± 6.4	6.2 ± 6.1	6.4 ± 6.3
PM	7.9 ± 8.1	7.1 ± 7.3	6.1 ± 5.4	6.6 ± 5.1
PL	7.3 ± 6.1	6.1 ± 5.6	6.0 ± 4.8	5.8 ± 5.0
**Physiological**				
AMPM	2.7 ± 2.2	2.1 ± 1.7	2.1 ± 1.7	2.3 ± 2.5
AM	3.1 ± 3.6	2.4 ± 3.3	2.2 ± 2.6	2.5 ± 3.3
PM	3.5 ± 4.4	3.1 ± 3.5	3.2 ± 4.0	3.2 ± 3.2
PL	2.9 ± 2.5	2.8 ± 2.2	3.0 ± 2.2	2.8 ± 1.9

Note: Data are presented as mean ± SD. None of the groups differed from placebo in daily stress response, psychological or physiological (*p *> 0.05).

### 3.3. PANAS

Age, weight, and sex were not significant predictors for PA nor NA (*p* > 0.05). There was a significant effect in the AM group for PA at the end of the cessation period (*p* = 0.003; *d* = 1.68), in which it continued to increase. The AMPM group showed a decrease in PA at the end of the cessation period (*p* = 0.009; *d* = 0.16). For NA, none of the conditions differed from placebo, AM (*p* = 0.454; η^2^ = 0.011), PM (*p* = 0.774; η^2^ < 0.001), and AMPM (*p* = 0.569; η^2^ = 0.002). Figure 4 contains PANAS results.

**Figure 4.**
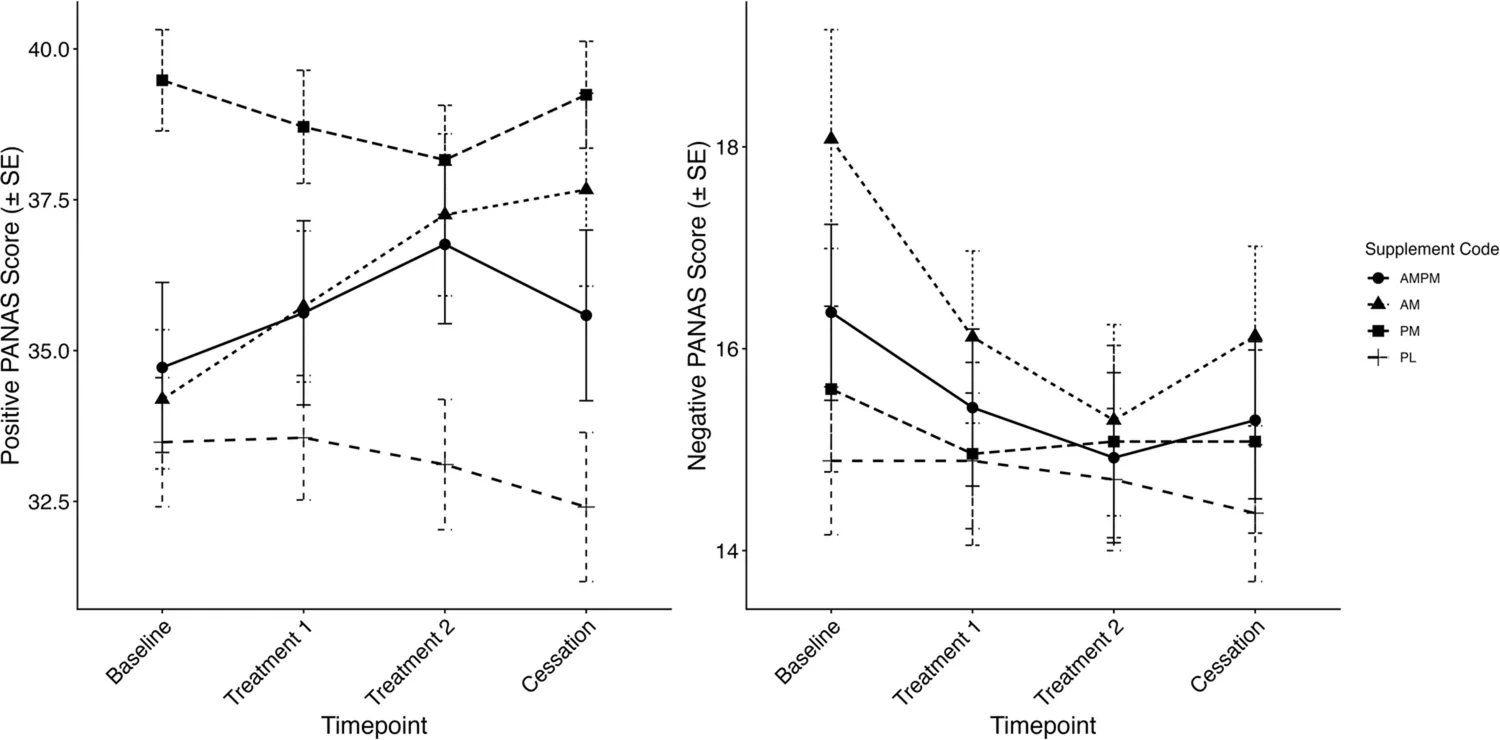
Group means ± standard error at each timepoint for both subscales of the positive and negative affect schedule scale. There was a significant effect in the AM group at the end of the cessation period (*p* = 0.003; d = 1.68), in which it continued to increase. The AMPM group showed a decrease in positive affect at the end of the cessation period (*p* = 0.009; d = 0.16). None of the conditions reached significance for negative affect as compared to the placebo group (*p* > 0.05).

### 3.4. PROMIS

Age, weight, and sex were not significant predictors for either PROMIS sleep disturbance nor sleep-related impairment (*p* > 0.05). None of the conditions reached significance for PROMIS sleep disturbances, AM (*p* = 0.088; η^2^ = 0.112), PM (*p* = 0.478; η^2^ = 0.018), and AMPM (*p* = 0.099; η^2^ = 0.088). The AM group showed a decrease in sleep-related impairments at treatment week 2 (*p* = 0.032, *d* = 2.74). The AMPM group had an increase in sleep-related impairments at the end of the cessation period (*p* = 0.038, *d* = −2.32). Figure 5 contains both sleep-related impairment and sleep disturbances.

**Figure 5.**
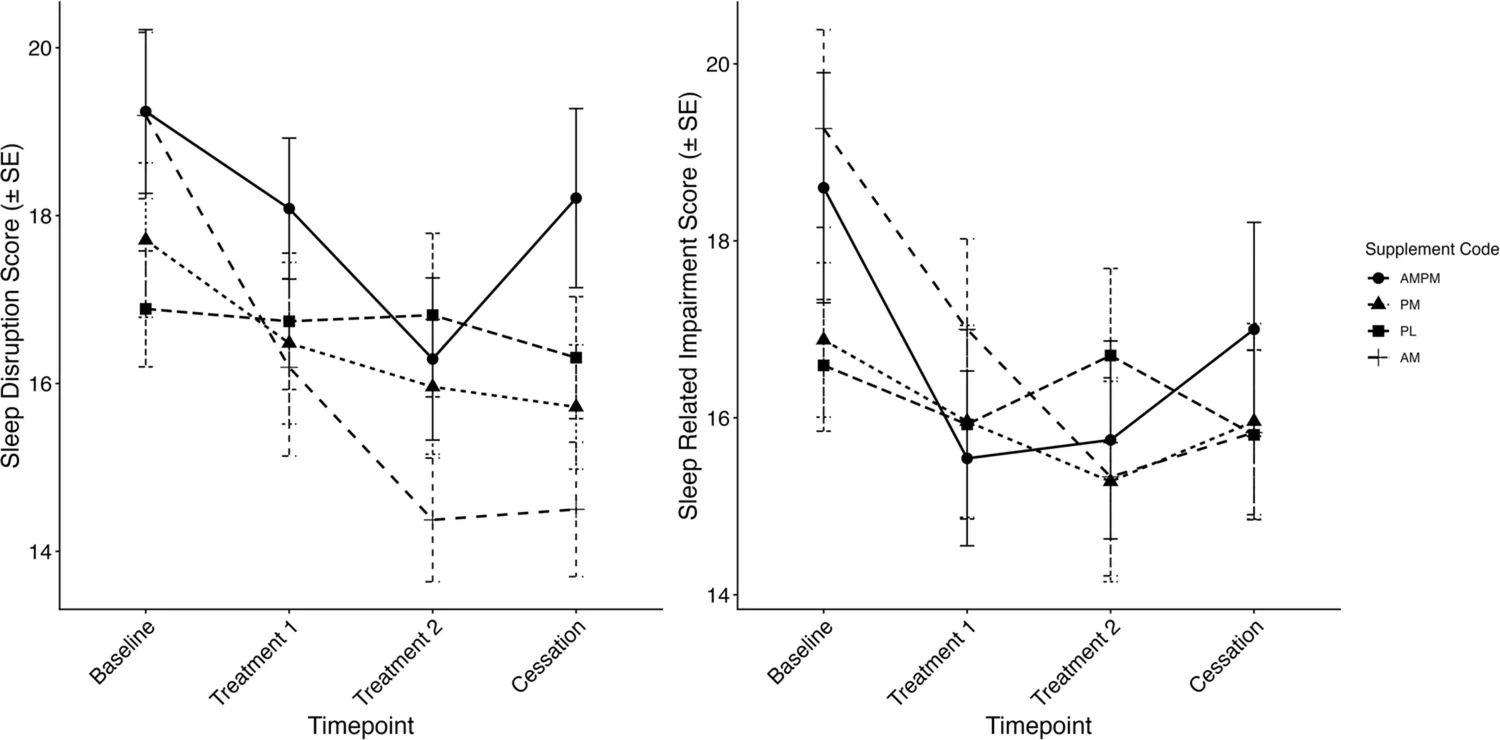
Group means ± standard error at each timepoint for patient reported outcome measurement information system sleep disturbances and sleep-related impairments. None of the groups reached significance for sleep disturbances (*p* > 0.05). The AM group showed a decrease in sleep-related impairments at treatment week 2 (*p* = 0.032, d = 2.74). The AMPM group had an increase in sleep-related impairments at the end of the cessation period (*p* = 0.038, d = −2.32).

### 3.5. Exploratory measures

#### 3.5.1. Pittsburgh sleep quality index

There were no time (*p* = 0.230, η^2^ = 0.01), group (*p* = 0.372, η^2^ = 0.03) or interaction (*p* = 0.259, η^2^ = 0.04) effects for the global PSQI score. No time (*p* = 0.352, η^2^ < 0.001) or group (*p* = 0.379, η^2^ = 0.03) main effects were found for subjective sleep quality. However, there was a significant interaction effect observed for subjective sleep quality (*p* < 0.001, η^2^ = 0.20). Follow-up pairwise comparisons indicated that subjective sleep quality was significantly improved in the AM group compared with the AMPM group (mean difference = 0.66 AU, *p* < 0.001, *d* = 1.45), with no observed differences between any other group (*p* > 0.05). A group main effect on subjective sleep latency was observed (*p* = 0.033, η^2^ = 0.08), but there were no significant post hoc comparisons (*p* > 0.05), nor time or interaction effects (*p* > 0.05). No other effects reached significance (*p* > 0.05). Table 4 presents the global PSQI and subscales at each timepoint for each group.

**Table 4.** Pittsburgh sleep quality index subscales by group and time.

Group	Baseline	Cessation	Time *p*-value (η^2^)	Group *p*-value (η^2^)	Interaction *p*-value (η^2^)
**Global PSQI**					
AMPM	4.3 ± 2.0	4.4 ± 1.7	0.23 (0.01)	0.37 (0.03)	0.26 (0.04)
AM	4.1 ± 1.9	3.3 ± 1.6			
PM	3.8 ± 1.9	3.8 ± 1.8			
PL	4.4 ± 1.9	4.3 ± 2.0			
**Subjective sleep quality**					
AMPM	1.0 ± 0.5	1.4 ± 0.7	0.35 (<0.01)	0.38 (0.03)	<0.001* (0.20)
AM	1.2 ± 0.6	0.7 ± 0.5			
PM	1.1 ± 0.6	1.0 ± 0.4			
PL	1.1 ± 0.5	1.0 ± 0.5			
**Sleep latency**					
AMPM	0.9 ± 0.9	0.8 ± 0.7	0.08 (0.03)	0.03* (0.08)	0.77 (0.01)
AM	0.6 ± 0.8	0.4 ± 0.6			
PM	0.5 ± 0.7	0.4 ± 0.6			
PL	0.9 ± 0.8	0.9 ± 0.9			
**Sleep duration**					
AMPM	0.2 ± 0.4	0.3 ± 0.5	0.89 (<0.01)	0.63 (0.02)	0.33 (0.03)
AM	0.3 ± 0.7	0.3 ± 0.5			
PM	0.2 ± 0.4	0.2 ± 0.4			
PL	0.4 ± 0.7	0.3 ± 0.5			
**Sleep efficiency**					
AMPM	0.1 ± 0.3	0.0 ± 0.0	0.08 (0.02)	0.26 (0.02)	0.26 (0.02)
AM	0.0 ± 0.0	0.0 ± 0.0			
PM	0.0 ± 0.0	0.0 ± 0.0			
PL	0.0 ± 0.2	0.0 ± 0.0			
**Sleep disturbances**					
AMPM	1.1 ± 0.4	1.2 ± 0.4	0.55 (<0.01)	0.24 (0.04)	0.70 (0.01)
AM	1.0 ± 0.3	1.0 ± 0.3			
PM	1.2 ± 0.4	1.2 ± 0.4			
PL	1.1 ± 0.4	1.2 ± 0.4			
**Daytime dysfunction**					
AMPM	0.8 ± 0.8	0.6 ± 0.7	0.09 (0.03)	0.57 (0.02)	0.72 (0.01)
AM	1.0 ± 0.6	0.8 ± 0.7			
PM	0.7 ± 0.6	0.6 ± 0.6			
PL	0.7 ± 0.6	0.7 ± 0.5			

Note: Data are presented as mean ± SD. *Denotes *p *< 0.05. PSQI = Pittsburgh sleep quality index. Subjective sleep quality was significantly improved in the AM group compared with the AMPM group (*p *< 0.001, *d *= 1.45). A group main effect in subjective sleep latency was revealed (*p *= 0.033, η^2^ = 0.08), but there were no significant post-hoc comparisons (*p *> 0.05).

#### 3.5.2. Objective sleep measures

Age, weight, and sex were not significant predictors for any outcomes (*p* > 0.05). No effects were observed for SQI, sleep efficiency, sleep latency, sleep duration, total sleep time, wake transitions, and average heart rate (*p* > 0.05).

The AM group had decreased WASO time (*p* = 0.047; η^2^ = 0.020) as compared to the placebo group across the intervention. The AM group (*p* = 0.049; d = 0.62) and PM group (*p* = 0.009; d = 0.94) had an increase in WASO after treatment week 2. The AMPM group had a decrease in WASO following the cessation week (*p* = 0.026; d = 0.003). Table 5 contains all objective sleep measure results.

**Table 5.** Objective sleep metrics by group and time.

Group	Baseline	Treatment 1	Treatment 2	Cessation	Group	Baseline	Treatment 1	Treatment 2	Cessation	
**Sleep quality index**					**Total sleep time (hrs)**					
AMPM	56.1 ± 16.8	57.6 ± 16.9	57.2 ± 16.9	56.3 ± 15.9	AMPM	6.4 ± 0.5	6.3 ± 0.6	6.4 ± 0.6	6.2 ± 0.9	
AM	47.0 ± 16.8	47.4 ± 15.5	47.8 ± 18.6	47.0 ± 17.3	AM	6.3 ± 0.6	6.2 ± 0.9	6.0 ± 0.7	5.9 ± 0.8	
PM	52. 7 ± 15.0	57.2 ± 16.7	54.6 ± 16.0	54.5 ± 15.7	PM	6.3 ± 0.7	6.1 ± 0.8	6.2 ± 0.8	6.1 ± 0.8	
PL	53.7 ± 18.3	54.3 ± 18.0	51.6 ± 17.4	55.0 ± 16.6	PL	6.5 ± 0.7	6.4 ± 0.7	6.4 ± 0.7	6.3 ± 0.7	
**Efficiency**					**Wake after sleep onset (hrs)**					
AMPM	86.4 ± 5.6	86.2 ± 4.1	86.2 ± 5.1	87.2 ± 3.7	AMPM	0.78 ± 0.20	0.79 ± 0.20	0.83 ± 0.29	0.79 ± 0.23	
AM	85.2 ± 4.1	86.2 ± 4.2	85.3 ± 6.1	85.4 ± 5.7	AM	0.90 ± 0.26	0.80 ± 0.22	0.85 ± 0.32	0.86 ± 0.33	
PM	85.9 ± 4.6	87.0 ± 4.2	85.2 ± 5.3	86.9 ± 5.7	PM	0.82 ± 0.28	0.73 ± 0.27	0.93 ± 0.28	0.77 ± 0.33	
PL	86.7 ± 4.5	86.7 ± 4.5	87.0 ± 4.8	86.6 ± 4.4	PL	0.78 ± 0.26	0.82 ± 0.32	0.77 ± 0.29	0.87 ± 0.34	
**Latency**					**Wake transitions**					
AMPM	8.2 ± 6.2	8.7 ± 4.3	9.5 ± 4.7	9.3 ± 5.8	AMPM	11.4 ± 2.3	11.0 ± 2.8	11.1 ± 3.0	11.7 ± 2.7	
AM	8.0 ± 5.8	9.7 ± 5.4	8.8 ± 5.8	8.6 ± 7.0	AM	12.2 ± 3.1	11.2 ± 2.6	11.4 ± 2.7	11.3 ± 3.4	
PM	9.4 ± 5.8	8.3 ± 8.6	8.0 ± 6.5	8.5 ± 6.1	PM	11.4 ± 3.2	10.6 ± 3.3	11.5 ± 3.1	11.2 ± 3.9	
PL	8.6 ± 6.6	7.6 ± 6.0	8.5 ± 6.0	7.7 ± 7.2	PL	10.6 ± 3.3	10.9 ± 3.7	11.0 ± 3.5	11.9 ± 4.2	
**Duration (hrs)**					**Heart rate averages (bpm)**					
AMPM	7.2 ± 0.5	7.1 ± 0.7	7.1 ± 0.7	6.9 ± 0.9	AMPM	59.7 ± 9.3	59.5 ± 7.9	58.8 ± 7.6	59.7 ± 8.4	
AM	7.1 ± 0.6	7.0 ± 0.6	6.8 ± 0.6	6.7 ± 0.8	AM	57.5 ± 8.3	56.9 ± 7.5	55.1 ± 7.8	58.0 ± 7.9	
PM	7.0 ± 0.8	6.8 ± 1.0	7.0 ± 0.8	6.9 ± 0.8	PM	58.9 ± 8.4	59.1 ± 8.2	56.3 ± 8.3	57.2 ± 6.8	
PL	7.2 ± 0.7	7.2 ± 0.8	7.2 ± 0.7	7.1 ± 0.8	PL	58.3 ± 5.6	58.9 ± 6.2	57.6 ± 5.9	58.2 ± 5.1	

Data are presented as mean ± SD. The AM group had decreased WASO time (*p *= 0.047; η^2^ = 0.020) as compared to the placebo group. The AM group (*p *= 0.049; *d *= 0.62) and PM group (*p *= 0.009; *d *= 0.94) had an increase in WASO after treatment week 2. The AMPM group had a decrease in WASO following the cessation week (*p *= 0.026; *d *= 0.003).

#### 3.5.3. Safety and compliance

Two male participants from the AM group were withdrawn from the study due to possible study-related adverse events. One participant reported mild gastrointestinal distress in the form of diarrhea after the third day of supplementation. The second participant reported moderate dizziness and mild muscle weakness following two days of supplementation. Both participants were removed from the study within 12 h of reporting symptoms, and symptoms were resolved within 48 h of discontinuing use. Neither required hospitalization nor medical action. No other potentially study-related adverse events were reported. Gastrointestinal distress is considered a common side effect, and dizziness or vertigo has been reported in prior studies of ashwagandha and *R. rosea* [53,54].

From TP 1 to TP 2, product consumption by pill count was 98.5% and was 97.5% as accounted for by daily diaries. For the total 2-week intervention, overall compliance was 99.7% as determined by pill counts and 94.6% for the 22 individuals whose compliance was calculated from daily study diaries. Two individuals fell between 70% and 80% compliance at TP 3 and were re-educated on study compliance.

## 4. Discussion

The present study evaluated the effects of morning adaptogen supplementation, evening sleep-focused supplementation, and their combined administration on subjective and objective sleep outcomes, perceived stress, and mood in healthy physically active adults. The primary finding was that morning supplementation with ashwagandha and *R. rosea* (AM group) produced the most consistent and notable improvements as compared to the placebo product. There were positive effects for perceived stress, PA, sleep-related impairments, and exploratory measures of sleep quality and WASO. The AMPM group had less favorable perceived stress, PA, sleep-related impairments, and exploratory subjective sleep quality than the AM group following the cessation week. Furthermore, evening supplementation (PM group) was associated with a decrease in WASO.

A potential explanation for these findings is that modulation of daytime stress physiology may be a key driver of perceived sleep quality, perhaps as much or even more so than directly targeting sleep with supplementation. However, this is speculative given that direct physiological measurement was not included in this study. Importantly, the multi-ingredient blend used in the present study has not previously been investigated, and prior literature demonstrating pronounced effects has largely focused on stressed or sleep-disturbed populations. Therefore, the present results extend prior work by demonstrating that, in adults without clinically significant stress or sleep dysfunction, daytime adaptogen supplementation may improve perceived stress and sleep quality and positive affect, while the addition of PM supplementation (MgT, L-theanine, and apigenin) may not provide additional benefits for select sleep-related outcomes.

The positive findings for the AM group are consistent with prior literature on adaptogens in that several trials report reductions in PSS scores following supplementation with ashwagandha or *R. rosea* [19,55], whereas others report no significant differences compared with placebo [56,57]. Further research is needed on ashwagandha, as many studies reporting reductions in PSS have used stressed samples or chronic interventions. Remenapp et al. [56] also observed a significant reduction in cortisol, however, the effects were inconsistent with subjective measures. The use of a healthy sample, as in the present study, gives further evidence of the efficacy of ashwagandha and *R. rosea.* While the PSS has been widely used in adaptogen research, the DSRS has been less frequently applied but was included in the present study to capture potential short-term fluctuations in stress, given its daily assessment structure.

Although some studies have reported rapid reductions in stress within one week of *R. rosea* supplementation [19,58], these investigations were open-label and conducted in stressed populations, limiting direct comparability. Collectively, these findings underscore that inconsistencies in the literature and disagreements between PSS and DSRS results may be driven by differences in population stress levels and intervention duration.

Interestingly, during the cessation week, the AM group showed an increase in PA, while the AMPM group showed a decrease. While this is in line with prior work reporting mood improvements following *R. rosea* supplementation, because these effects were observed post-cessation period, it cannot necessarily be assumed they were entirely due to study product effects. Cropley et al. [19] observed improvements across multiple mood domains in stressed adults following 200 mg of *R. rosea* twice a day, although the study employed an open-label design. The absence of significant changes in mood during the 2-week treatment period may be attributable to the use of a healthy, non-stressed, and non-mood-disturbed sample, which may limit the capacity to detect mood-related improvements. Given the well-established relationship between affective state and perceived sleep quality, future studies should evaluate whether individuals with elevated stress, mood disturbance, or poorer baseline affect profiles demonstrate greater responsiveness [59].

### 4.1. Subjective sleep measures

The AM group showed a decrease in sleep-related impairments following the two-week treatment period. This sustained improvement in subjective sleep in the AM group aligns with the hypothesis that reducing daytime stress load may influence sleep more so than targeting sleep alone. Despite no significant changes in global PSQI scores, a robust group-by-time interaction was observed for the PSQI subscale of subjective quality, with the AM group demonstrating significantly greater improvements than the AMPM group. Due to the PSQI global score reflecting seven domains of sleep, improvements confined primarily to perceived sleep quality may have been insufficient to shift the composite score. The timing of questionnaire administration may have influenced this observation, as the PSQI is validated for monthly use and was only completed the week before baseline and after the cessation period, limiting its sensitivity to detect changes during the intervention. The potential decrease in subjective sleep quality in the AMPM group may indicate an interaction effect between the AM and PM supplements or simply a titration of effects after supplement cessation.

The findings in the AM group align with prior work examining ashwagandha and *R. rosea*. In an open-label trial of individuals with chronic fatigue, after eight weeks of 400 mg/day of *R. rosea,* PSQI scores fell from 8.0 ± 3.1 to 3.7 ± 2.2, falling below the threshold typically used to classify impaired sleep (≥5) [49]. Similarly, ten weeks of 300mg/day of ashwagandha supplementation significantly reduced PSQI scores in individuals with insomnia [16], with comparable findings later observed in both patients with insomnia and healthy adults following eight weeks of supplementation [17]. In addition to sleep-specific findings, both ashwagandha and *R. rosea* have been shown to reduce fatigue in stressed or fatigued populations [20,57]. Thus, reductions in daytime fatigue may influence subjective perception of sleep quality, even in the absence of measurable changes in objective sleep.

### 4.2. Objective sleep measures

The only significant objective sleep measure was WASO, which improved in the AM group throughout the intervention. Subjective sleep measures are strongly influenced by stress, affect, and cognitive arousal, whereas objective measures may remain relatively stable in the absence of clinically meaningful sleep pathology [60]. In healthy populations without clinical sleep pathology, objective sleep data often demonstrate limited variability, reducing the likelihood of detecting measurable change in short-duration interventions [61]. Device-related exclusions reduced the sample size for objective sleep analyses, limiting the statistical power.

### 4.3. PM supplement blend

Taken together, these findings suggest that morning-only supplementation with ashwagandha and *R. rosea* was sufficient to elicit significant improvements across multiple outcome measures, whereas evening-only supplementation was insufficient. The absence of superiority in the AMPM group may reflect non-additive or diminishing returns when multiple agents targeting overlapping stress and arousal pathways are combined. Although not directly assessed in the present study, it is conceivable that modulation of daytime stress was the primary driver of perceived improvements in sleep [62].

Based on prior research and accurate dosages for each individual supplement, there is a possibility of an interaction among the ingredients in the PM group. For example, Noah et al. [43] reported a negative interaction in stressed adults, whereby a combination of magnesium (150 mg), L-theanine (50 mg), *R. rosea* (222 mg), and B vitamins increased PSQI daytime dysfunction scores without improving stress as assessed by the Depression Anxiety Stress Scale. Moreover, despite limited peer-reviewed evidence, anecdotal reports suggest that magnesium L-threonate may produce energizing or focusing effects, possibly related to its role in cognitive biochemical pathways [63]. Finally, low baseline sleep disturbances in this healthy sample may have constrained the magnitude of expected outcomes. As such, reductions in daytime stress may have had a greater impact on subjective sleep than supplementation directly targeting sleep quality. The observed pattern of multiple outcome measures showing time-dependent improvement is consistent with the well-documented placebo response in sleep interventions and underscores the importance of a rigorously controlled experimental design.

Several limitations should be acknowledged. First, stress outcomes were assessed solely through self-report questionnaires, which can capture perceived stress but may not align with physiological indices of stress and are susceptible to recall bias. Future research should incorporate objective biomarkers to further elucidate underlying mechanisms. Second, technical issues with the sleep device resulted in missing objective sleep data, reducing statistical power for those analyses. Further, due to manufacturer recommendations and as this specific combination has not been studied before, a two-week supplementation period was chosen, which may not have been long enough to elicit greater changes. As there were two participants who experienced adverse events, future research should keep this in mind and include safety markers. Lastly, due to the first wave of 22 participants being shipped a variable amount of study products due to a change in packaging (but not dosing) during manufacturing, their compliance could not be tracked through pill counts and instead had to rely on study diary reporting. Despite these limitations, this study possesses notable strengths. The inclusion of both subjective and objective sleep measures provides a comprehensive assessment of sleep outcomes. Additionally, whereas much of the adaptogen literature focuses on populations experiencing clinical stress or sleep disturbances, the present investigation extends these findings to healthy adults, where use of these supplements is arguably more prevalent. In addition, inclusion of both separate and combined AM and PM supplementation allowed for unique insight into the potential mechanistic efficacy of these ingredients.

## 5. Conclusions

Overall, the findings indicate that morning supplementation with a blend of ashwagandha and *R. rosea* was associated with improvements in perceived stress, subjective sleep quality, and PA in healthy adults after 14 days of supplementation. The absence of significant changes in certain outcome measures in the present study likely reflects the use of a generally healthy sample or a shorter intervention duration.

Collectively, these findings suggest that morning supplementation with ashwagandha and *R. rosea* may be a strategy to improve stress, sleep quality, and mood in healthy, physically active adults, without the need for more complex daytime/nighttime supplementation protocols. Future research should extend these findings by directly comparing responses between healthy and high-stress or poor sleep populations, examining longer intervention durations, and incorporating both subjective and objective outcome measures to further clarify the conditions under which these supplements may be most efficacious.
